# PROTOCOL: The impact of agricultural mechanisation on women's economic empowerment: A mixed‐methods systematic review

**DOI:** 10.1002/cl2.1334

**Published:** 2023-06-23

**Authors:** Edoardo Masset, Suchi Kapoor Malhotra, Neha Gupta, Ratika Bhandari, Howard White, Heather MacDonald, Ranjitha Puskur, Niyati Singaraju, Hugh Sharma Waddington

**Affiliations:** ^1^ London School of Hygiene and Tropical Medicine London UK; ^2^ Campbell South Asia Delhi India; ^3^ Carleton University Ottawa Canada; ^4^ International Rice Research Institute New Delhi India

## Abstract

This is the protocol for a Campbell systematic review. The main objective of the review is to answer the following questions: What is the impact of mechanisation on agriculture? What is the impact of mechanisation on women's economic empowerment? The study will review the impact of mechanisation on labour demand and supply, land and labour productivity, farmers' incomes, health and women's empowerment. All literature will be considered, including nonintervention studies and studies not reporting gender‐disaggregated results.

## 1 BACKGROUND

Agriculture is the primary source of income for 60% of women in Oceania, Southern Asia and Sub‐Saharan Africa, and for 80% of women in the world's least‐developed countries (LDCS) (UN Women, 2015). Women produce 60–70% of the food in most developing nations, and account for over half of all worldwide food production (Food and Agriculture Organization [FAO], 2011). They represent a large fraction of the agricultural labour force, though there are large disparities across countries (Palacios‐Lopez et al., 2017).

Despite women's major contributions to agriculture, gender disparities (in terms of vulnerability, access to resources, and production) characterise their agricultural activities (Huyer, 2016). Evidence suggests that women could boost their agricultural outputs by 20%–30% if they had access to the same productive agricultural resources as men (Food and Agriculture Organization FAO, 2011). Global data show that women's agricultural productivity per unit of land is lower than men's (e.g., Goldstein & Udry, 2008; Peterman et al., 2011) due to lower access to inputs (e.g., fertiliser, improved seeds), limited time availability, and lack of land property rights and human capital, including education and agricultural knowledge (Kilic et al., 2015).

Bilateral and international agencies promote agricultural interventions to improve yields, earnings, time availability, and food security, which contribute to women's empowerment (USAID, 2017). They have highlighted mechanisation as a critical tool for closing the gender‐productivity gap in agriculture while improving women's empowerment and advancing broader welfare outcomes.

Mechanisation is a process by which human labour substitutes animal power, tools or machines. It increases labour productivity and farmers' incomes, as well as land productivity by enhancing cropping intensity and timeliness in the completion of crop operations (Binswanger, 1986; Pingali, 2007).

The impact of mechanisation on women will depend on the prevailing gender division of labour in the area under study (Doss, 2001). In several countries, it is common for agricultural tasks, crops and labour relations to differ by gender. Differences are shaped by differential control over and access to resources, and by cultural norms. Women are frequently confined to low‐skilled labour and conventional farming methods to grow subsistence crops. The gender gap is compounded by a lack of access to mechanisation, and a lack of inclusion in the design of machinery that is tailored to women's needs.

Differences vary across countries and are changing over time. This implies that the gender impact of mechanisation is difficult to predict and challenging to disentangle from the impact of other factors after its implementation (Doss, 2001). A summary of the gender impact of mechanisation cannot be conducted by abstracting from the specific context in which that mechanisation is taking place.

Embedded structural impediments, sociocultural norms and gender norms all contribute to gender inequities in smallholder agriculture. For example, in many contexts women are not considered able to operate complex machines. This is equally true in terms of technological development and application. The present priorities, beliefs and norms around both agricultural systems and gender are reflected in technological design and dissemination (USAID, 2017). Several technologies have been developed without careful consideration of the needs of various end users, resulting in low adoption rates.

There is ample evidence on the positive impact of mechanisation on agricultural productivity and growth, but evidence of its impact on disadvantaged men and women is scant and mixed (Daum, 2020). It is often believed that mechanisation disproportionally favours male farmers as they have better access to large farms and engage in tasks that are more easily substituted by machines. However, the labour‐saving element of mechanisation may also favour women farmers, particularly if the saved time can be more efficiently employed in other activities—provided that alternative employment opportunities are available. The evidence in this regard is limited and mixed (Daum, 2020).

Even less evidence is available on the impact of mechanisation on health. Mechanisation can reduce most strenuous forms of agricultural work and free women's time for their employment in economic activities or health seeking behaviours.

Recognising this evidence gap, the Consultative Group on International Agricultural Research (CGIAR) GENDER (Generating Evidence and New Directions for Equitable Results) platform commissioned the present review, which employed mixed methods to shed light on the impacts of mechanisation interventions, specifically those on women's empowerment, by reviewing both quantitative and qualitative studies. The confidence rating of studies in the review will help to identify high‐quality impact and process evaluations that might have the potential to inform future policies in the area.

## 2 OBJECTIVES

This review is concerned with the impact of agricultural mechanisation. Interventions to promote agricultural mechanisation are included in the review, as are studies examining the effects of its adoption in the absence of any intervention.

Agricultural mechanisation is a process by which human work is substituted with alternative forms of energy, such as animal power, fossil energy, or renewable energy, along the entire agricultural value chain (Malabo Montpellier Panel, 2018). The UN Food and Agriculture Organisation FAO defines mechanisation as the use of tools, implements and machinery to achieve agricultural production (Clarke, 1997). There has been a resurgence of interest in mechanisation in agricultural research and development, with a renewed focus on equity and sustainability (Fischer et al., 2018).

Mechanisation produces various positive effects. It minimises hard labour, reduces drudgery, and alleviates labour shortages. There is some evidence suggesting that it supports women's empowerment by expanding their assets and time availability (Theis et al., 2018). However, women have limited access to mechanisation and are often excluded from mechanisation interventions, as they are typically designed and implemented by men who do not consider women as a target group (Lahai et al., 1999). Extension systems tend to view women as welfare recipients rather than as active players in agricultural development (Food and Agriculture Organization [FAO], 2011). Mechanisation can also affect women negatively, for example by reducing employment among female agricultural labourers, or by reducing their bargaining power within the household.

Understanding the impact of agricultural mechanisation and interventions promoting it, as well as their role in women's economic empowerment, can better inform agricultural policies.

## 3 METHODS

In this section we discuss the role of mechanisation in agriculture and its impact on women's welfare and empowerment. The analysis of the impact of mechanisation in agriculture is one of the oldest in development literature. Two key readings of this literature—Ruttan's *Induced Innovation and Agricultural Development* (1977) and Binswanger's *Agricultural Mechanisation: A Comparative Historical Perspective* (1986)—were published in the second issue of *Food Policy* and the first issue of the W*orld Bank Research Observer*, respectively. Our framework for understanding the impact of mechanisation on agriculture draws on these two sources, as well as reviews by Pingali (2007) and Daum (2020).

**Figure 1:**
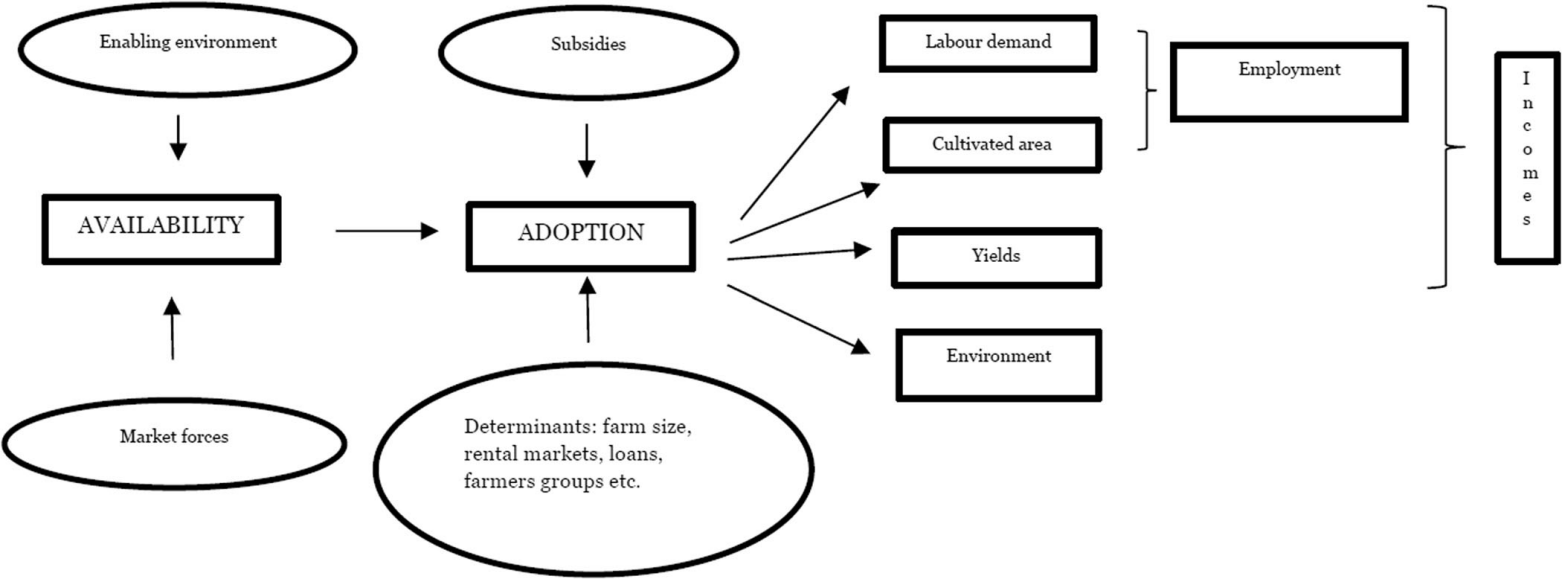
A theory of change of mechanisation.

The diagram in Figure 1 presents our theory of change of mechanisation, which identifies three steps: availability, adoption and impact. The steps are sequential, meaning that mechanisation can have an impact only if the tools are available and adopted. The circles in Figure 1 list the factors that affect availability and adoption.

Agricultural technologies are developed in response to market forces. This is the main tenet of the theory of induced innovation, which is often used to explain patterns of technical change (Ruttan, 1977). Market forces lead to the development of technologies that use relatively less of the factor that is less abundant, and therefore more expensive. A labour‐scarce country is more likely to develop labour‐saving technologies, while a land‐scarce country is more likely to develop and adopt land‐saving technologies. In land‐abundant and labour‐scarce countries, prices will lead farms to substitute labour with machines. The public sector can contribute to this process in various ways: by facilitating the transfer of innovation from other countries, adapting them to the characteristics of the physical and social context, or by supporting innovations that are tailored to specific farmers' needs.

Once a technology has been developed and made available, it must be adopted and used. There is a significant amount of literature on factors affecting the adoption of new technologies. In the case of mechanisation, the following are determinant factors: mechanical technology is indivisible and often too costly to be affordable to farmers operating small farms. Loans may allow the purchase of new technologies, though small farmers do not have easy access to them. Therefore, large farms are more likely to adopt mechanical technology as they have the means to purchase it and to use it in an economically efficient way.

Accessibility issues can be addressed through rental markets or through the formation of co‐operatives. If rental markets for mechanised tools are feasible, or if famers can join co‐operatives that share mechanical inputs, small farmers can then access new technologies without loans. There are successful examples of custom hiring centres for mechanised tools, including centres run by women (FAO, 2021) (examples from South Asia are discussed in Lokesh et al., 2018 and Srinivasarao et al., 2013). However, rental markets and co‐operatives are not available everywhere (Daum, 2020).

Operation and maintenance of mechanical tools also require specialised knowledge and skills. Poor farmers are often at a disadvantage due to a lack of education and skills required to operate complex machines. Maintenance and repair services are major bottlenecks to the efficient use of mechanical tools, although examples of small farmers and women trained to use and provide maintenance are emerging (e.g., Kawarazuka et al., 2018; Polar et al., 2017).

When it comes to the use of mechanical technology, a distinction is made between ‘power‐functions’—such as land preparation (ploughing and tilling), threshing, chopping, transport, and water‐pumping—which require strength and physical energy, and ‘control functions’—such as seeding, planting, harvesting, weeding, and spraying—which require attention and judgement (Pingali, 2007). Machines excel at performing power functions, although the development of artificial intelligence may change this in the future, and it is possible to imagine new machinery excelling in control functions. Farmers tend to first adopt technologies that perform power‐intensive functions, and to substitute labour with control‐function machines only when wages are high and labour becomes unavailable (Pingali, 2007).

Historically, governments have promoted the adoption of mechanical technology through subsidies. This has often been carried out irrespective of local market or geographic conditions, in such a way that the results of these policies have rarely been successful (Binswanger, 1986).

Once the technology is adopted and used, it has a series of impacts on labour use, land use, factor productivity, the environment, and possibly health. First, mechanisation reduces labour demand, at least in the short term. Mechanical tools are designed to replace physical work and their immediate effect is a reduction in the use of labour per unit of land. The reduction in labour demand applies equally to own‐labour and hired labour.

In the short term, agricultural labourers will be negative affected by mechanisation as labour demand and employment decrease. However, in the long term the farmed area may increase, with mechanisation potentially leading to a net increase in labour demand. The final result will depend on the availability of land and the demand elasticity of goods produced (Binswanger, 1986). An increase in agricultural production requires a sufficiently elastic demand to be absorbed or exported to international markets.

Mechanisation can also have positive impact on yields, for example through changes in cropping intensity. Finally, it has been observed that mechanisation could have some negative environmental effects due to its consumption of fossil fuels, promotion of monocultures and land erosion (Daum, 2020).

The overall impact of mechanisation on incomes will differ between adopting farmers and agricultural labourers. Farmers will benefit from an increase in labour productivity, expansion of cultivated land, and increasing yields. Agricultural labourers can be negatively affected by a reduction in employment, although the expansion of cultivated areas and the increase in wages can mitigate this effect. The environmental effects, such as erosion and loss of diversity, will negatively affect everyone.

### 3.1 Gender impact of agricultural mechanisation

Figure 1 illustrated the impact of mechanisation on farmers and agricultural labourers independently of farmers' genders. However, the impact of mechanisation on men and women will be different for at least three reasons: first, agricultural technology is often designed by men, for men (Ragasa, 2012). It is argued that the mechanical technology available, with its focus on power functions rather than control functions, is designed to replace men's labour rather than women's.

Second, women are less likely to adopt new technologies due to various constraints such as low access to loans, complementary inputs, information and specialised education, as well as restrictive social norms (Vemireddy & Choudhary, 2021). Third, men and women often perform different agricultural tasks, even cultivating different plots of land and different crops. As a result, the impact of mechanisation will be different for men and women (Doss, 2001).

In relation to the last point, however, it should be noted that there is great heterogeneity across countries and contexts in the extent to which men and women perform different agricultural tasks. As a result, the impact of mechanisation on women is not easily predictable or transferable from one context to another (Doss, 2001). Moreover, any distribution of tasks is subject to change, including endogenous changes produced by the introduction of new technologies. For example, a mechanical tool that decreases women's labour or increases their productivity may lead men to shift their resources towards the tasks and crops supported by the new technology.

**Figure 2:**
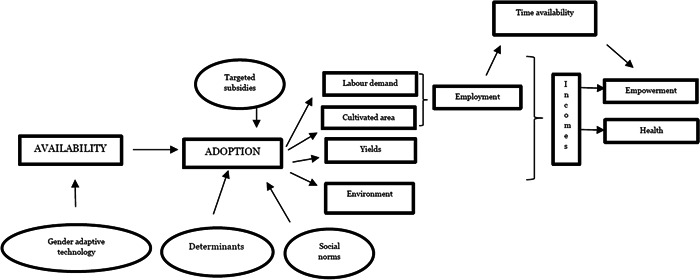
Impact of mechanisation on women.

Figure 2 shows the expected impacts of mechanisation on women's welfare. The diagram reproduces that of Figure 1, but adds additional gender‐specific outcomes and determining factors. As previously mentioned, much agricultural technology is designed to replace male, rather than female, labour. However, technological developments have allowed for the development of machines that replace control functions traditionally carried out by women, such as weeding and seeding.

Women have limited access to new technologies, facing various constraints ranging from insufficient levels of education or training to efficiently operate machines, lack of access to loans that allow for the purchase of machines, to adversarial social norms. The ability to buy or rent tools is affected by farm size, and plots managed by women tend to be small and unprotected by property rights. Machines alone cannot improve production in the absence of other complementary inputs (such as land, fertiliser and hired labour) that women may find difficult to obtain. In other words, mechanical technology is more likely to be adopted by farms managed by men, in such a way that the impacts of mechanisation on women occur more often as a secondary effect.

However, women farmers are unequivocally positively affected by mechanisation. The use of mechanical tools reduces labour in specific tasks and increases the size of cultivated land. This leads to an increase in labour productivity and income, which in turn can improve women's economic empowerment and health outcomes.

Mechanisation can also reduce ‘time poverty’. The double burden of hard agricultural labour and domestic work negatively affects women's education, participation in social activities, and healthcare‐seeking behaviour (Hyde et al., 2020). A reduction in time poverty provides more time for healthcare‐seeking behaviour—in addition to engaging in more profitable economic activities or in training to acquire knowledge and skills. Provided that economic opportunities are available, this newly available free time may produce additional income.

The impact of mechanisation on health is rarely investigated. Women in rural areas are involved in strenuous and repetitive work, which leads to cumulative stress and physical disorders leading to various disabilities. A reduction in drudgery is likely to produce a reduction in work‐related physical impairments. In addition, health effects in the form of better nutrition and access to healthcare for the entire family may arise from an increase in income.

Changes in income and time availability can improve women's empowerment in various ways. Additional income may provide access to properties and assets. It may allow expenditure for education and family planning. It may delay marriages and increase mobility and independent agency. An increase in women's income may also augment their bargaining power within the household and increase women's ability to make independent life choices.

The impact on women engaged in agricultural labour, however, is less obvious. If mechanisation reduces the demand for hired labour, and agricultural labourers are predominantly female, then women labourers will be negatively affected. A reduction in wage incomes will also have negative consequences on empowerment and health. If mechanisation expands the size of cultivated land and increases labour demand and wages, then women labourers will be positively affected.

Finally, the impact on the women in households of male adopters is impossible to predict. If males adopt mechanical tools, household income may increase, but this will not necessarily improve women's welfare, and empowerment can even deteriorate. The same is true for the impact on time availability. Women's free time may increase, but it could also decrease if, for example, an expansion in the cultivated area requires an increase in their labour.

The following is a summary of the potential impacts of mechanisation on women. Notice that these impacts are hypothetical rather than observed, as they are based on theoretical assumptions and a literature review, and can be considered testable hypotheses:

- Women are less likely to adopt mechanical technology due to constraints such as: access to credit and savings, insurance mechanisms, information and skills and property rights; and various transaction costs (e.g., Suri & Udry, 2022);
- Women farmers are positively affected in terms of income, time availability, health and economic empowerment;
- Women labourers can be adversely affected in terms of employment, income and empowerment, although this can be compensated by an expansion of farmed area and increased wages;
- Women and men agricultural labourers will be differently affected depending on the agricultural task that is being mechanised (mechanisation primarily affected power functions in the early stages of mechanisation, but both gender roles in agriculture and the mechanical tools available have changed); and
- Women in the households of male adopters can be affected positively or negatively. The impact will be highly context specific and impossible to predict.

## 4 PRIOR REVIEWS

There are several literature reviews that summarise the impacts of agricultural mechanisation, including those on gender. For example, the excellent review by Daum (2020) examined literature on mechanisation against nine common propositions (or ‘myths’) such as: ‘mechanisation increases unemployment’, and ‘smallholder farmers cannot benefit from mechanisation’. Peterman and colleagues (2010) and Ragasa (2012) are examples of studies that reviewed empirical literature on gender difference regarding access and adoption of agricultural technologies, including mechanised tools. However, these reviews, although very informative, do not employ a systematic approach. The literature search is not exhaustive, the quality of the studies is not critically assessed, and the results are not summarised with the use of meta‐analysis techniques.

We found three studies that do employ a systematic review methodology, and cover some aspects included in our review. Aduwo and colleagues (2019) conducted a systematic review of the literature to identify factors that produce gender differences in technology adoption, and found that access to resources (land, labour, capital, nonfarm income, inputs and extension services), educational level, distance to market, decision‐making power, participation in associations, and norms and beliefs are key factors, among others. The authors covered an important aspect of our review—access to agricultural technology—but were not specifically focused on mechanised technology.

Hemming and colleagues (2018) systematically reviewed literature on the effectiveness of input subsidies to promote adoption, productivity and incomes, where inputs included mechanised tools. They found a limited impact of interventions on the outcomes considered. The review however has a narrow focus on subsidies and covers support to mechanised inputs in a marginal way.

Vemireddy and Choudhary (2021) systematically reviewed the literature on factors affecting women's adoption of labour‐saving technology (i.e., mechanised tools) and their impact. They identified factors affecting adoption—such as access to extension services and membership of groups and organisations—and the impacts of mechanisation on labour use and time availability. However, the evidence was not reported against a coherent theory of how the intervention works, and the authors did not use meta‐analytic methods.

Our assessment of the existing reviews on mechanisation and gender is that most existing reviews did not employ rigorous systematic review approaches; they covered limited and narrow aspects (e.g., factors determining adoption); they did not review the impact of mechanisation along a coherent theory of how the intervention works, and they did not use meta‐analysis methods to summarise the quantitative studies. Our review proposes to carry out all of the above.

Aduwo, O. E., Aransiola, J. O., Ikuteyijo, L. O., Alao, O. T., Deji, O. F., Ayinde, J. O., & Oyedele, D. J. (2019). Gender differences in agricultural technology adoption in developing countries: a systematic review. *African Vegetables Forum, 1238*, 227–238.

Daum, B. R. (2020). Agricultural mechanisation in Africa: myths, realities, and a research agenda. *Global Food Security, 26*.

Hemming, D. J., Chirwa, E. W., Dorward, A., Ruffhead, H. J., Hill, R., Osborn, J., & Phillips, D. (2018). Agricultural input subsidies for improving productivity, farm income, consumer welfare and wider growth in low‐and lower‐middle‐income countries: a systematic review. *Campbell Systematic Reviews*, *14*(1), 1–153.

Peterman, A., Behrman, J., & Quisumbing, A. (2011). *A review of empirical evidence on gender differences in non‐land agricultural inputs, technology, and services in developing countries* (ESA Working Paper 11‐11). FAO.

Ragasa, C. (2012). *Gender and institutional dimensions of agricultural technology adoption*:

*A review of literature and synthesis of 35 case studies*. [Selected Poster]. August 18–24 International Association of Agricultural Economists (IAAE) Triennial Conference, Foz do Iguaçu, Brazil.

Vemireddy, V., & Choudhary, A. (2021). A systematic review of labour‐saving technologies: Implications for women in agriculture. *Global Food Security*, *29*, 100541.

## 5 OBJECTIVES

The review will address the following questions:

1. **What is the impact of mechanisation on agriculture?** We will review the impact of mechanisation on labour demand and supply, productivities, incomes, health and the environment. All literature will be considered, including non‐intervention studies and studies not reporting gender‐disaggregated results.
2. **What is the impact of mechanisation on women's economic empowerment and well‐being?** We will review the impact of mechanisation on women's outcomes. All the literature identified above will be considered, provided the results are disaggregated by gender.
3. **What are the impacts of mechanisation interventions and gender‐responsive interventions?** We will review the impacts found by evaluations of interventions promoting mechanisation, including those specifically designed to positively affect women by increasing their productivity, reducing drudgery, or more generally empowering them. From the literature identified above, we will classify interventions and review their effectiveness separately, with a particular focus on economic empowerment outcomes.
4. **What factors support gender‐responsive mechanisation interventions?** Impacts of agricultural mechanisation on women are shaped by the prevailing gender division of labour, access to resources, decision‐making and cultural norms. We will identify the supporting and derailing factors of mechanisation interventions promoting women's economic empowerment. To answer this question, we will adopt a theory‐based approach and will review the ability of the interventions to achieve the intended outcomes along the causal chain, at the same time identifying contextual factors that favour or hinder this process. Qualitative process evaluations will provide the contextual evidence needed to explain why the interventions work or do not work. We will be able to answer questions such as: which mechanisation technologies are adopted by women and why? How do gender norms and constraints limit or enhance women's use and adoption of mechanised tools? How do intra‐household dynamics affect women's control and use of mechanised tools? Do custom hiring services enable greater adoption of agricultural machinery among women?
5. **Building a mid‐level theory of the impact of mechanisation?** A secondary goal of our review is the development of a mid‐level theory of the impact of mechanisation in agriculture. Current available theories fall into one of two categories: first, there are very general theories, such as the induced innovation theory, which explain in abstract terms the development and the impact of mechanisation. They highlight very general laws or tendencies but are unable to explain specific project outcomes. Second, there are highly specific and specialised theories of change, which explain how an intervention will work in a specific context. These theories of change are very detailed but their lessons do not easily transfer to other interventions in other settings. Our goal is to formulate a theory that refines the theories of change presented in the sections above—in short, a theory that is not too general and not too specific, whose mechanisms apply to a wide variety of contexts.

## 6 CRITERIA FOR CONSIDERING STUDIES FOR THIS REVIEW

Studies will be included in the review if they meet the following selection criteria for population, outcomes and study design, as well as intervention.

## 7 POPULATION

The population of interest under this review are men and women engaged within the agricultural sector in low‐ and middle‐income countries (LMICs), as defined by the World Bank categorisation.

## 8 INTERVENTION

In relation to Research Questions 1 and 2, we will consider mechanisation broadly defined as the replacement of human or animal work with mechanical tools. Mechanisation will be reported in the literature in the form, for example, of mechanised planting and harvesting, postharvest processing, or tractor use. An aggregate indicator of mechanisation in the form of agricultural capital stock in the context of cross‐country or cross‐sectional analyses will also be considered. To address Research Question 2 in particular, we will review studies reporting the results of studies exclusively of women, or disaggregated by the gender of farmers, heads of households, or subgroups that can be easily assigned to a particular gender (e.g., women agricultural labourers).

In relation to Research Questions 3 and 4, we will consider studies reporting the results of interventions designed to promote the use of mechanised tools in general, and by women in particular. Interventions promoting mechanisation will include:

- Research and development initiatives promoting mechanisation;
- The establishment of an economic and trade environment that supports mechanisation;
- Subsidised provision of mechanical tools;
- Training and extension services promoting mechanisation; and
- Loans, rental markets and other arrangements promoting the use of mechanised tools.

Interventions promoting women's use of mechanised tools will include:

- The establishment of a research environment that favours the development or the adaptation of technologies to suit women's needs;
- Provision and promotion of tools that are specifically designed to meet women's needs;
- Subsidised tools specifically targeted to women, including donated tools;
- The provision, or increased access and affordability, of tools directly or through farmers' Organisations, self‐help groups and co‐operatives; and
- The removal of constraints that prevent women's adoption of technology, such as credit, skills formation and social norms.

## 9 OUTCOMES

We define four types of welfare outcomes, which will be further refined after reviewing the literature:

1. The highest‐level outcomes consist of indicators of economic empowerment. There is a huge variety in the use of empowerment indicators across studies and contexts. They tend to fall into one of four categories (Buvinic, 2017): (1) composite indices of various dimensions of economic empowerment; (2) self‐reported indicators of women's agency within the household (i.e., the ability to make autonomous decisions about use of income and resources); (3) psychological tests of autonomy and agency; or (4) achievement indicators (e.g., participation in economic activities, access to markets, ability to make economic decisions, bargaining power and control over resources). All reported indicators of economic empowerment will be considered.
2. The second level includes health impacts, such as a reduction in disability burdens, an increase in healthcare‐seeking behaviour, and improvement in child nutrition and health.
3. The third level includes a series of positive economic effects brought about by mechanisation. These include wage rates of male and female agricultural workers, employment (including job losses and displacement), the use of own‐labour, the allocation of time on tasks within the family, the use of hired labour, yields, cultivated areas, crops, income, time availability off‐work, and the acquisition of new knowledge and skills.
4. Finally, at the lower tier of outcomes we will consider participation indicators as basic preconditions for any higher‐level outcome. These will include participation in the promoted interventions, technology adoption, and group membership.

Some of the outcomes in Table 1 can only be measured at the household level, such as income and land productivity. They can however be disaggregated by the gender of the household head, which is a customary practice in the literature. This approach has some limitations. First, many women are involved in agriculture, including operating and managing their own plots or other related activities, while also being members of a male‐headed households. The impacts of interventions on these women are ignored by simply comparing male‐ and female‐headed households.

**Table 1:** Outcome indicators.

**Outcomes**	**Indicators**
Empowerment	Indices of economic empowerment
	Agency indicators of decision‐making on use of resources
	Psychological tests of autonomy and agency
	Achievement indicators such as leadership positions in groups, ability to make economic decisions, access to markets, bargaining power, and control over resources
Health effects	Disability
	Healthcare‐seeking behaviour
	Health outcomes
	Child nutrition
Economic effects	Male and female wages
	Employment (displacements)
	Labour productivity
	Use of own and hired labour
	Cultivated area and yields
	Time use and drudgery
	Income and expenditure
Participation	Participation in interventions
	Group membership
	Technology adoption

Second, households are not female headed by chance, and have special characteristics that are not representative of the wider female population. For example, they might be richer or poorer. Third, household change composition and female‐headed households are not permanent or immutable. Despite these limitations, results disaggregated by the gender of the household head is sometimes all that is available.

Some outcomes of Table 1 are observed at the individual level. Individual‐level indicators include empowerment indicators, employment status, time allocation to different activities and domestic tasks, nutritional status, and empowerment indicators. Finally, some outcomes are observed at the individual level for each household member and are still relevant to our review, particularly children's health outcomes.

## 10 STUDY DESIGNS

Few experimental studies will be available to answer Research Questions 1 and 2 regarding the general impacts of mechanisation in agriculture. We will consider quasi‐experimental studies and econometric studies that employ rigorous methods of counterfactual analysis as well as methods employing standard econometric methods, and hypothetical modelling simulations. These will include:

- Quasi‐experimental methods such as: difference in difference, matching, instrumental variables, regression discontinuity, synthetic controls;
- Panel fixed‐effect regressions, including cross‐country regression analyses; and
- Computable general equilibrium models and other modelling methods building hypothetical counterfactuals.

To answer Research Question 3, we will include studies with an experimental or quasi‐experimental design. Eligible designs include those in which the authors used a control or comparison group and in which one of the following is true:

- Participants were randomly assigned (using a process of random allocation, such as a random number generation);
- A quasi‐random method of assignment was used and pretreatment equivalence information is available regarding the nature of group differences (and groups generated are essentially equivalent);
- Participants are non‐randomly assigned but matched on pre‐tests and/or relevant demographic characteristics (using observables, or propensity scores) and/or according to a cut‐off on an ordinal or continuous variable (regression discontinuity design); or
- Participants were non‐randomly assigned, but statistical methods were used to control for differences between groups (e.g., using multiple regression analysis, including difference‐in‐difference, cross‐sectional [single differences], or instrumental variables regression).

No restriction will be placed on the duration of follow up.

To answer Research Question 4, we will include qualitative process evaluations/qualitative studies of interventions promoting women's use of mechanical tools. We will include evaluations or qualitative studies of an eligible intervention discussing design and implementation issues.

### 10.1 Stakeholder consultation

This review topic has been commissioned by the CGIAR GENDER Platform. In consultation with the staff of the Platform, we will form a small advisory group of researchers and practitioners.

## 11 SEARCH STRATEGY

We have devised a search string to capture the studies relevant to our research questions. The search string is based on our review PICOs. We will search the following databases:

### 11.1 Electronic searches

#### 11.1.1 Databases

World Agricultural Economics and Rural Sociology Abstracts (on CABI), Scopus (limit to Social Sciences), Web of Science Core Collection, Gender Studies (on Ebscohost), Greenfile (on Ebscohost), Econlit (on Ebscohost), Business Source Complete (on Ebscohost), IBSS (on Proquest), PAIS (on Proquest), PsycINFO (on Proquest), Medline (on Web of Science) (1950–present), CAB ABstract (on CABI), AfricaBib and the World Bank.

In addition to a traditional, manual database search, we will conduct a machine learning‐assisted search in EPPI‐Reviewer beta version (Microsoft Academic data set/Open Alex). Microsoft Academic data set, like Google Scholar, is a comprehensive repository of research articles containing 250 million bibliographic records. The results from the two approaches to database searching will be combined.

#### 11.1.2 Searching other resources

We will screen the bibliographies of included studies and existing reviews for eligible studies. We will also hand‐search the table of contents for the last five years for the journals listed below (Table 2).

**Table 2:** List of journals.

S. No	Title
1	*Journal of Rural Studies*
2	*Ecological Economics*
3	*World Development*
4	*Sustainability*
5	*Environmental Research*
6	*Society & Natural Resources*
7	*Food Security*
8	*Food Policy*
9	*Development*
10	*Review of Development Economics*
11	*PLOS One*
12	*Women's Studies International Forum*
13	*Gender, Technology, and Development*
14	*Agricultural Economics*
15	*European Review of Agricultural Economics*
16	*Agriculture and Human Values*
17	*European Review of Development Research*
18	*Forum for Development Studies*
19	*Gender, Place, and Culture*
20	*International Journal of Agricultural Sustainability*
21	*Journal of Integrative Agriculture*
22	*Journal of Gender Studies*
23	*Agroforestry Systems*
24	*Agricultural Systems*
25	*Agricultural and Food Economics*
26	*Sustainability Science*
27	*Gender and Society*
28	*Journal of Gender, Agriculture and Food Security (Agri‐Gender)*
29	*Gender and Development*
30	*American Journal of Agricultural Economics*
31	*Journal of Development Economics*

In addition, we will the search relevant websites listed in Table 3. We will snowball to other websites identified in these searches, systematically documenting each website searched (website, URL, date, any filters or search strings used, and studies identified for screening).

**Table 3:** List of websites.

S. No	Website
1	AgriProFocus
	https://agriprofocus.com/intro
2	Bill & Melinda Gates Foundation
	https://www.gatesfoundation.org/
3	CGIAR
	https://www.cgiar.org/
4	Donor Committee for Enterprise Development
	https://www.enterprise-development.org/
5	International Labour Organisation
	https://www.ilo.org/global/lang--en/index.htm
6	International Livestock Research Institute
	https://www.ilri.org/
7	Netherlands Development Organisation
	https://snv.org/
8	The Food and Agriculture Organisation
	https://www.fao.org/about/en/
9	The International Fund for Agricultural Development
	https://www.ifad.org/en/
10	The Department for International Development
	https://www.gov.uk/government/organisations/department-for-international-development</bold>
11	The International Food Policy Research Institute
	https://www.ifpri.org/
12	The Swiss Agency for Development and Cooperation
	https://www.eda.admin.ch/sdc
13	The United States Agency for International Development
	https://www.usaid.gov/
14	World Agroforestry
	https://www.worldagroforestry.org/
15	World Bank
	https://www.worldbank.org/en/home
16	UN Women
	https://www.unwomen.org/en

## 12 DATA COLLECTION AND ANALYSIS

### 12.1 Selection of studies

Screening of studies for inclusion and exclusion will be undertaken in two stages using EPPI‐Reviewer 4. In the preliminary stage, a title and abstract screening will be carried out. The second stage will encompass a full text screening. Both stages of screening will be performed by two independent researchers against predefined inclusion criteria for the review, with a third‐party arbitrator in case of disagreement.

### 12.2 Data extraction and management

For the impact and process evaluation, we will use a standardised data extraction form to extract required data from all studies that meet our inclusion criteria. Data extraction from each study will include contextual and geographical information, population, study design and method, intervention type and outcomes type, and subcategory. Two researchers will carry out the data extraction for each study Disagreements would be resolved through discussion with a third‐party reviewer.

### 12.3 Assessment of risk of bias in included studies

Confidence in the study findings of all studies included in the review will be assessed using a risk‐of‐bias tool for effectiveness studies, and a critical appraisal tool for primary studies, developed by the Campbell Collaboration Secretariat. Coding for critical appraisals will be carried out by two independent reviewers.

The tool contains critical dimensions of the evaluation. Each of these is marked as high, medium, or low. The overall score uses the ‘weakest link in the chain’ principle. Hence, confidence in study findings can only be as high as the lowest rating given to the nine critical items in qualitative/process evaluation.

### 12.4 Unit of analysis issues

The primary unit‐of‐analysis for the quantitative data within the studies of interest will usually be the individual. It is expected that these studies will report data at the programme level, reporting aggregate data for all women in the programme.

Multiple papers or reports based on the same study or data will be treated as a single case for purposes of this review, which fits with our proposed approach to mixed‐methods analysis (described below) in which the unit of analysis is the case, not the paper. That is, a paper report will only be considered as a separate case if the research sample does not include study participants included in any other coded study.

Where there are multiple papers, we will select the most complete reference if all relevant information is available in a single source. However, if multiple reports each provide different information (e.g., different outcomes or different subgroups), then the data from these reports will be coded as a single case.

### 12.5 Statistical procedures and conventions

Most impact indicators of mechanisation, such as income or input use, are continuous. Some outcomes, including the adoption of mechanisation, will be reported as dichotomous variables. To perform the meta‐analysis, we will use odds ratios for dichotomous variables and Hedge's *g* for continuous variables (as Hedge's *g* is preferred over Cohen's *d* for small samples, which is expected to be the case for the number of studies included in this review). Odds ratios will be computed via the available information for other effect sizes found in primary studies, such as proportions, percentages, raw frequencies, regression coefficients, chi‐square and marginal distributions. All effect size calculations will be performed using the Campbell online effect size calculator (Wilson, n.d.).

Given the variety of settings and of interventions considered, our meta‐analysis will employ a random effects model. We will start the analysis using a restricted maximum likelihood estimator, and will test with different estimators to assess the sensitivity of the results. We will adjust standard errors using Knapp‐Hartung adjustments to reduce the chances of false positives.

### 12.6 Assessment of heterogeneity

Heterogeneity between effect sizes studies will be assessed by reporting the *I* ^2^ and *τ* ^2^; however, our key approach to addressing heterogeneity will consist of calculating and reporting prediction intervals, and interpreting results based on the prediction intervals.

Forest plots will be generated for visual representation of pooled effect size. The causes of heterogeneity, if any, will be identified by moderator analysis. Separate forest plots will be presented for important moderators such as geographic study area and type of intervention (type of mechanisation).

### 12.7 Multiple reports of the same outcome

It is common in the economics literature to report many effect sizes. This primarily happens for three reasons: using different model specifications, reporting results for subgroups, and measuring the same outcomes using different indicators. We will treat such instances based on the reason for multiple reports as follows:

- Model specification: We will select the effect size from the model preferred by the authors when reporting the results. If the authors stated no obvious preference for the specification model, we will select the model that in our view is less likely to be biased.
- Subgroup analysis: We will combine subgroups that are not overlapping and independent into a single estimate (by taking the weighted average and adjusting the standard error accordingly). We will not use multiple results for different subgroups, and will rely on estimates for the whole sample.
- Different indicators: When the same construct (e.g., income) is measured using different metrics (e.g., per capita income, per capita expenditure, and wealth asset), we will average the effect sizes and the standard errors.

Depending on the complexity of the data extracted (i.e., the number and variety of effects sizes reported in each study), we will also employ a multi‐level meta‐analysis to allow multiple effect sizes within the study, which are mutually dependent. The analysis will be carried out using the *metafor* package in R.

### 12.8 Intention‐to‐treat versus treatment‐of‐the‐treated outcome measures

Differential attrition will be reported during the coding stage for all quantitative studies, as it is one of the items in the critical appraisal tool.

Where attrition is high, it matters whether the reported effect size is intention‐to‐treat or treatment‐of‐the‐treated. The two should not be combined into a single meta‐analysis. Where a study reports a treatment‐of‐the‐treated effect size, it will be converted to intention‐to‐treat if the data are available to do so, so that the study can be used in the overall analysis of intention‐to‐treat effects.

### 12.9 Treatment of publication bias

Publication‐selection bias will be assessed for the primary outcomes by constructing a funnel plot). The funnel plot will be used for a trim‐and‐fill analysis and the calculation of Egger's test. (Egger et al., 1997).

### 12.10 Planned moderator analyses

We will carry out a priori planned moderator analyses. *Post hoc* moderator analyses may be used depending on the analysis of patterns of heterogeneity in the data.

Based on the theory of change presented in the section on ‘how the intervention might work’, we identified the following moderators for the meta‐analysis:

- Geographic location of the intervention: There is a fundamental difference in the process of mechanisation, particularly in Africa and in Asia
- Mechanisation tools adopted: There is a fairly regular sequential pattern of mechanisation across countries, whereby mechanised water pumps are adopted first, followed by tractors, and finally by harvesters and other more specialised machines. The type of machine (pump, tractor, and other) will be employed.
- Type of intervention: Interventions promoting mechanisation can be broadly subdivided into interventions enabling an environment that increases farmers' access to mechanisation, and interventions that more proactively provide subsidies or mechanised tools directly. Whether the intervention enables the environment or provides mechanised tools will be another key moderator of the analysis.

### 12.11 Mixed‐methods analysis (treatment of qualitative research)

Carvalho and White (2004) identified various ways in which qualitative data may be used in an analysis of quantitative data. These ways are similar to those identified in the Cochrane Handbook, which states that ‘qualitative evidence synthesis (commonly referred to as QES) can add value by providing decision makers with additional evidence to improve understanding of intervention complexity, contextual variations, implementation, and stakeholder preferences and experiences’.

This review adopts that approach (i.e. combining qualitative data with a quantitative meta‐analysis) within the framework of a theory‐based systematic review (White, 2018). This approach, which has similarities with the framework synthesis approach (Booth & Carroll, 2015; takes the intervention as the unit of analysis, rather than the individual study. Different studies may contribute findings at different stages of the causal chain. Specifically, qualitative data can be:

- Integrated with quantitative data to elaborate the causal chain (i.e., the different causal mechanisms within the theory of change). For example, there may be a large gap between intention‐to‐treat and treatment‐of‐the‐treated effect sizes due to high attrition or women failing to avail themselves of mechanised agricultural techniques. Qualitative data are usually best placed to understand barriers and facilitators to participation;
- Used to confirm, enrich and illustrate the findings of quantitative data. Quotes from women supporting these causal mechanisms add colour to the report, strengthening confidence in the effect as one that operates through the posited causal mechanism;
- Used to explain study findings. The theory‐based systematic review approach uses the funnel of attrition to recognise the fact that effect sizes shrink as they move along the causal chain from outputs and intermediate outcomes to final outcomes;
- The relevant factors in mechanisation interventions may poorly use of mechanisation services for various reasons, weak links in the causal chain;
- Contradictory to or challenging the intended causal mechanisms, possibly leading to a counter‐theory (Carvalho & White, 2004); and
- Merged with findings from quantitative analysis into a single set of implications for policy and practice.

Quantitative data are indicated as Qt and qualitative data as Ql. Quantitative data refers to both effect sizes and factual quantitative data, such as participation rates. As shown in the table, we will test the consistency of the data with various theories identified in the theory of change.

Table 4 shows the theory‐based systematic review framework which is used for both horizontal and vertical synthesis (White, 2018). Vertical synthesis involves summarising the evidence across all cases, which is how systematic reviews are usually performed, especially for quantitative analysis of effects. In the case of qualitative data, vertical synthesis is a thematic analysis, in which common themes are identified across studies.

**Table 4:** Theory‐based systematic framework.

Case 1	Horizontal synthesis
Case 2	
—	
Case n	
Vertical synthesis	Overall synthesis

Horizontal synthesis summarises across a case—which may be performed in narrative reviews—however the difference here is that the data for an intervention may be drawn from more than one study. The overall synthesis combines both, though it may also contain a separate overall synthesis by subgroup. The overall synthesis approach, drawing on both horizontal and vertical synthesis, ‘tells the story’ of whether the intervention works, for whom, under what circumstances, and why.

## ROLES AND RESPONSIBILITIES

Content: Ranjitha Puskur and Edoardo Masset are responsible for content. Edoardo Masset is also the technical lead for the review.

Systematic review methods: Edoardo Masset, Hugh Sharma Waddington, Howard White and Suchi Kapoor Malhotra are responsible for ensuring that satisfactory systematic review methods are used.

Statistical analysis: Edoardo Masset will lead on statistical analysis and Suchi Kapoor Malhotra will be assisting.

Qualitative data analysis: Suchi Kapoor Malhotra is responsible for performing qualitative data analysis.

Information retrieval: Edoardo Masset and Suchi Kapoor Malhotra are responsible for information retrieval, based on searches designed by Heather MacDonald, Information Retrieval Specialist with Campbell IDCG.

## POTENTIAL CONFLICT OF INTEREST STATEMENT

Howard White is CEO of the Campbell Collaboration. He has no role in the editorial decisions regarding this review.

There are no other conflicts of interest.

## PRELIMINARY TIMEFRAME

Note, if the protocol or review is not submitted within 6 and 18 months of title registration, respectively, the review area is opened up for other authors.

- Date you plan to submit a draft protocol: 1 December 2021
- Date you plan to submit a draft review: 10 August 2022

## Supporting information

Supporting information.
