# Supplementary material for: PROTOCOL: The impact of agricultural mechanisation on women's economic empowerment: A mixed‐methods systematic review
Source: Campbell Syst Rev. 2023 Jun 23;19(3):e1334. doi: 10.1002/cl2.1334 (PMC10288359; doi:10.1002/cl2.1334)
Supplement: Supplementary file 1 — Supporting information. [file CL2-19-e1334-s001.docx]

# Appendix A: Search terms

| Scopus Database  search strategy | Hits |
| --- | --- |
| ((gender* OR woman OR women OR mother* OR female* OR man OR men OR male* OR worker* OR labourer* OR farmer* OR "hired hand*" OR farmworker* OR fisherman OR fishermen OR fisherwoman OR fisherwomen OR operator* OR entrepreneur* OR family OR household OR input* OR smallholder* OR landowner* OR "land owner*")) AND ((((mechaniz* OR mechanise* OR mechanisa* OR motor* OR pneumatic OR hydromechanic* OR mechanic* OR engine OR engines OR hydraulic OR diesel OR robot* OR "remote control*" OR automat* OR electronic* OR electric* OR computeri* OR "articifical intelligence" OR "power operated" OR powered OR ((labor OR labour) NEAR/3 saving) OR machinery OR (technolog* NEAR/3 (adopt* OR mobili* OR accept* OR embrac* OR "take up" OR adapt* OR diffusion OR acquisition OR innovat* OR implement* OR access* OR improved)) OR reaper* OR harvester* OR tractor* OR cultivator* OR harrow* OR plow* OR plough* OR roller* OR destoner* OR windrower* OR "stone picker" OR tiller* OR rototill* OR coulter* OR planter* OR aerat* OR furrow* OR "cotton gin*" OR "cotton pick*" OR "seed clean*" OR "seed count*" OR drill* OR weeder* OR dibber* OR subsoiler* OR mower* OR "brush cutt*" OR pruning OR feeder* OR shear* OR transplanter* OR (logging NOT "data logging") OR chipping OR chipper OR bale OR baler* OR "tree shaker*" OR pump* OR "land leveler*" OR bulldozer* OR excavator* OR "trench cutter*" OR "feed process*" OR "feed mill*" OR incubator* OR hatcher* OR feller* OR skidder* OR delimber* OR sprayer* OR fogger* OR spreader* OR dispenser* OR sprinkler* OR hydroponic* OR "conveyor belt*" OR auger* OR binder* OR swather* OR huller* OR winnow* OR backhoe* OR loader* OR grinder* OR mixer* OR chillcur* OR thresher* OR "heat processing" OR "seed processing" OR "post harvest processing" OR "postharvest processing" OR "forage chopp*" OR sheller* OR shelling OR boiler* OR pasteurizer* OR pasteuriser* OR steamer* OR burner* OR "fish feeder*" OR trawl* OR sower* OR cultiseeder*) NEAR/8 (agricultur* OR agrarian OR agribusiness* OR agronom* OR husbandry OR farm OR farms OR farming OR smallhold* OR landown* OR "land own*" OR outgrower* OR pisciculture OR aquaculture OR breed* OR fish* OR "food produc*" OR floricultur* OR horticultur* OR silviculture* OR apiculture* OR aboricultur* OR forest* OR agroforestry OR agriforestry OR agroecolog* OR agrifood* OR agri-food* OR livestock OR crop OR crops OR harvest OR harvesting OR irrigate OR irrigation OR cultivate OR cultivation OR rice OR maize OR cassava OR taro OR coffee OR chicory OR coco* OR soya* OR tamarind OR dairy OR milk* OR sugar* OR "mung bean" OR chickpea OR barley OR millet OR rye OR sorghum OR wheat)) OR ((power OR smart OR moderni*) NEAR/2 (farm OR farming OR agriculture)) )) AND (("impact evaluation" OR "program* evaluation" OR "process evaluation" OR "random* control* trial*" OR "random* trial*" OR rct* OR ( random* NEAR/3 allocat* ) OR "instrumental variable*" OR "synthetic control" OR "intervention stud*" OR (experimental NEAR/1 (study OR design)) OR "quasi experiment*" OR "quasi-experiment*" OR dif-dif OR "double difference" OR difference-in-difference OR "difference in difference" OR "multiple regression" OR "multivariate regression" OR "multivariable regression" OR "bivariate regression" OR "statistical regression" OR "regression discontinuity*" OR "regression analysis" OR "statistical matching*" OR "propensity score matching" OR "covariate matching" OR "coarsened-exact matching" OR "propensity-weighted" OR inverse-probability-weighting OR "mixed method*" OR "cohort stud*" OR "cross sectional" OR "cross-sectional" OR "cohort analysis" OR "quantitative method*" OR "interrupted time series" OR ( before NEAR/5 after ) OR ( pre NEAR/5 post ) OR ( ( pretest OR "pre test" ) AND ( posttest OR "post test" ) ) OR ( "fixed effect*" NEAR/3 ( model OR estimation ) ) OR ( "random effect*" NEAR/3 ( model OR estimation ) ) OR ( ( quantitative OR "comparison group*" OR counterfactual OR "counter factual" OR counter-factual OR experiment* OR comparative ) NEAR/3 ( design OR study OR analysis)) OR (("semi-structured" OR semistructured OR prestructured OR pre-structured OR unstructured OR informal OR "in-depth" OR indepth OR "face-to-face" OR structured OR guide) NEAR/2 (interview* OR discussion* OR questionnaire*)) OR "focus group*" OR qualitative OR ethnograph* OR fieldwork OR "field work" OR "key informant" OR participatory OR "action research" OR "cooperative inquiry" OR "co-operative inquiry" OR case-stud* OR "case stud*" OR "community led" OR community-led OR barrier* OR facilitator* OR enabler* OR "panel data" OR (bivariate AND model) OR (multivariate AND model) OR (program* NEAR/3 (impact* OR assess*)) OR (gender* NEAR/3 (analysis OR approach*)) )) AND ((afghanistan OR albania OR algeria OR "american samoa" OR angola OR "antigua and barbuda" OR antigua OR barbuda OR argentina OR armenia OR armenian OR aruba OR azerbaijan OR bahrain OR bangladesh OR barbados OR belarus OR byelarus OR belorussia OR byelorussian OR belize OR "british honduras" OR benin OR dahomey OR bhutan OR bolivia OR "bosnia and herzegovina" OR bosnia OR herzegovina OR botswana OR bechuanaland OR brazil OR brasil OR bulgaria OR "burkina faso" OR "burkina fasso" OR "upper volta" OR burundi OR urundi OR "cabo verde" OR "cape verde" OR cambodia OR kampuchea OR "khmer republic" OR cameroon OR cameron OR cameroun OR "central african republic" OR "ubangi shari" OR chad OR chile OR china OR colombia OR comoros OR "comoro islands" OR "iles comores" OR mayotte OR "democratic republic of the congo" OR "democratic republic congo" OR congo OR zaire OR "costa rica" OR "cote d'ivoire" OR "cote d' ivoire" OR "cote divoire" OR "cote d ivoire" OR "ivory coast" OR croatia OR cuba OR cyprus OR "czech republic" OR czechoslovakia OR djibouti OR "french somaliland" OR dominica OR "dominican republic" OR ecuador OR egypt OR "united arab republic" OR "el salvador" OR "equatorial guinea" OR "spanish guinea" OR eritrea OR estonia OR eswatini OR swaziland OR ethiopia OR fiji OR gabon OR "gabonese republic" OR gambia OR "georgia (republic)" OR georgian OR ghana OR "gold coast" OR gibraltar OR greece OR grenada OR guam OR guatemala OR guinea OR "guinea bissau" OR guyana OR "british guiana" OR haiti OR hispaniola OR honduras OR hungary OR india OR indonesia OR timor OR iran OR iraq OR "isle of man" OR jamaica OR jordan OR kazakhstan OR kazakh OR kenya OR "democratic people's republic of korea" OR "republic of korea" OR "north korea" OR "south korea" OR korea OR kosovo OR kyrgyzstan OR kirghizia OR kirgizstan OR "kyrgyz republic" OR kirghiz OR laos OR "lao pdr" OR "lao people's democratic republic" OR latvia OR lebanon OR "lebanese republic" OR lesotho OR basutoland OR liberia OR libya OR "libyan arab jamahiriya" OR lithuania OR macau OR macao OR "macedonia (republic)" OR macedonia OR madagascar OR "malagasy republic" OR malawi OR nyasaland OR malaysia OR "malay federation" OR "malaya federation" OR maldives OR "indian ocean islands" OR "indian ocean" OR mali OR malta OR micronesia OR "federated states of micronesia" OR kiribati OR "marshall islands" OR nauru OR "northern mariana islands" OR palau OR tuvalu OR mauritania OR mauritius OR mexico OR moldova OR moldovian OR mongolia OR montenegro OR morocco OR ifni OR mozambique OR "portuguese east africa" OR myanmar OR burma OR namibia OR nepal OR "netherlands antilles" OR nicaragua OR niger OR nigeria OR oman OR muscat OR pakistan OR panama OR "papua new guinea" OR "new guinea" OR paraguay OR peru OR philippines OR philipines OR phillipines OR phillippines OR poland OR "polish people's republic" OR portugal OR "portuguese republic" OR "puerto rico" OR romania OR russia OR "russian federation" OR ussr OR "soviet union" OR "union of soviet socialist republics" OR rwanda OR ruanda OR samoa OR "pacific islands" OR polynesia OR "samoan islands" OR "navigator island" OR "navigator islands" OR "sao tome and principe" OR "saudi arabia" OR senegal OR serbia OR seychelles OR "sierra leone" OR slovakia OR "slovak republic" OR slovenia OR melanesia OR "solomon island" OR "solomon islands" OR "norfolk island" OR "norfolk islands" OR somalia OR "south africa" OR "south sudan" OR "sri lanka" OR ceylon OR "saint kitts and nevis" OR "st. kitts and nevis" OR "saint lucia" OR "st. lucia" OR "saint vincent and the grenadines" OR "saint vincent" OR "st. vincent" OR grenadines OR sudan OR suriname OR surinam OR "dutch guiana" OR "netherlands guiana" OR syria OR "syrian arab republic" OR tajikistan OR tadjikistan OR tadzhikistan OR tadzhik OR tanzania OR tanganyika OR thailand OR siam OR "timor leste" OR "east timor" OR togo OR "togolese republic" OR tonga OR "trinidad and tobago" OR trinidad OR tobago OR tunisia OR turkey OR "turkey (republic)" OR turkmenistan OR turkmen OR uganda OR ukraine OR uruguay OR uzbekistan OR uzbek OR vanuatu OR "new hebrides" OR venezuela OR vietnam OR "viet nam" OR "middle east" OR "west bank" OR gaza OR palestine OR yemen OR yugoslavia OR zambia OR zimbabwe OR "northern rhodesia" OR "global south" OR "africa south of the sahara" OR "sub-saharan africa" OR "subsaharan africa" OR "africa, central" OR "central africa" OR "africa, northern" OR "north africa" OR "northern africa" OR magreb OR maghrib OR sahara OR "africa, southern" OR "southern africa" OR "africa, eastern" OR "east africa" OR "eastern africa" OR "africa, western" OR "west africa" OR "western africa" OR "west indies" OR "indian ocean islands" OR caribbean OR "central america" OR "latin america" OR "south and central america" OR "south america" OR "asia, central" OR "central asia" OR "asia, northern" OR "north asia" OR "northern asia" OR "asia, southeastern" OR "southeastern asia" OR "south eastern asia" OR "southeast asia" OR "south east asia" OR "asia, western" OR "western asia" OR "europe, eastern" OR "east europe" OR "eastern europe" OR "developing country" OR "developing countries" OR "developing nation*" OR "developing population*" OR "developing world" OR "less developed countr*" OR "less developed nation*" OR "less developed population*" OR "less developed world" OR "lesser developed countr*" OR "lesser developed nation*" OR "lesser developed population*" OR "lesser developed world" OR "under developed countr*" OR "under developed nation*" OR "under developed population*" OR "under developed world" OR "underdeveloped countr*" OR "underdeveloped nation*" OR "underdeveloped population*" OR "underdeveloped world" OR "middle income countr*" OR "middle income nation*" OR "middle income population*" OR "low income countr*" OR "low income nation*" OR "low income population*" OR "lower income countr*" OR "lower income nation*" OR "lower income population*" OR "underserved countr*" OR "underserved nation*" OR "underserved population*" OR "underserved world" OR "under served countr*" OR "under served nation*" OR "under served population*" OR "under served world" OR "deprived countr*" OR "deprived nation*" OR "deprived population*" OR "deprived world" OR "poor countr*" OR "poor nation*" OR "poor population*" OR "poor world" OR "poorer countr*" OR "poorer nation*" OR "poorer population*" OR "poorer world" OR "developing econom*" OR "less developed econom*" OR "lesser developed econom*" OR "under developed econom*" OR "underdeveloped econom*" OR "middle income econom*" OR "low income econom*" OR "lower income econom*" OR "low gdp" OR "low gnp" OR "low gross domestic" OR "low gross national" OR "lower gdp" OR "lower gnp" OR "lower gross domestic" OR "lower gross national" OR lmic OR lmics OR "third world" OR "lami countr*" OR "transitional countr*" OR "emerging econom*" OR "emerging nation*")) | 5,798 |
| (gender* OR woman OR women OR mother* OR female* OR man OR men OR male* OR worker* OR labourer* OR farmer* OR "hired hand*" OR farmworker* OR fisherman OR fishermen OR fisherwoman OR fisherwomen OR operator* OR entrepreneur* OR family OR household OR input* OR smallholder* OR landowner* OR "land owner*") | 437,453 |
| (((mechaniz* OR mechanise* OR mechanisa* OR motor* OR pneumatic OR hydromechanic* OR mechanic* OR engine OR engines OR hydraulic OR diesel OR robot* OR "remote control*" OR automat* OR electronic* OR electric* OR computeri* OR "articifical intelligence" OR "power operated" OR powered OR ((labor OR labour) NEAR/3 saving) OR machinery OR (technolog* NEAR/3 (adopt* OR mobili* OR accept* OR embrac* OR "take up" OR adapt* OR diffusion OR acquisition OR innovat* OR implement* OR access* OR improved)) OR reaper* OR harvester* OR tractor* OR cultivator* OR harrow* OR plow* OR plough* OR roller* OR destoner* OR windrower* OR "stone picker" OR tiller* OR rototill* OR coulter* OR planter* OR aerat* OR furrow* OR "cotton gin*" OR "cotton pick*" OR "seed clean*" OR "seed count*" OR drill* OR weeder* OR dibber* OR subsoiler* OR mower* OR "brush cutt*" OR pruning OR feeder* OR shear* OR transplanter* OR (logging NOT "data logging") OR chipping OR chipper OR bale OR baler* OR "tree shaker*" OR pump* OR "land leveler*" OR bulldozer* OR excavator* OR "trench cutter*" OR "feed process*" OR "feed mill*" OR incubator* OR hatcher* OR feller* OR skidder* OR delimber* OR sprayer* OR fogger* OR spreader* OR dispenser* OR sprinkler* OR hydroponic* OR "conveyor belt*" OR auger* OR binder* OR swather* OR huller* OR winnow* OR backhoe* OR loader* OR grinder* OR mixer* OR chillcur* OR thresher* OR "heat processing" OR "seed processing" OR "post harvest processing" OR "postharvest processing" OR "forage chopp*" OR sheller* OR shelling OR boiler* OR pasteurizer* OR pasteuriser* OR steamer* OR burner* OR "fish feeder*" OR trawl* OR sower* OR cultiseeder*) NEAR/8 (agricultur* OR agrarian OR agribusiness* OR agronom* OR husbandry OR farm OR farms OR farming OR smallhold* OR landown* OR "land own*" OR outgrower* OR pisciculture OR aquaculture OR breed* OR fish* OR "food produc*" OR floricultur* OR horticultur* OR silviculture* OR apiculture* OR aboricultur* OR forest* OR agroforestry OR agriforestry OR agroecolog* OR agrifood* OR agri-food* OR livestock OR crop OR crops OR harvest OR harvesting OR irrigate OR irrigation OR cultivate OR cultivation OR rice OR maize OR cassava OR taro OR coffee OR chicory OR coco* OR soya* OR tamarind OR dairy OR milk* OR sugar* OR "mung bean" OR chickpea OR barley OR millet OR rye OR sorghum OR wheat)) OR ((power OR smart OR moderni*) NEAR/2 (farm OR farming OR agriculture)) ) | 80,690 |
| ("impact evaluation" OR "program* evaluation" OR "process evaluation" OR "random* control* trial*" OR "random* trial*" OR rct* OR ( random* NEAR/3 allocat* ) OR "instrumental variable*" OR "synthetic control" OR "intervention stud*" OR (experimental NEAR/1 (study OR design)) OR "quasi experiment*" OR "quasi-experiment*" OR dif-dif OR "double difference" OR difference-in-difference OR "difference in difference" OR "multiple regression" OR "multivariate regression" OR "multivariable regression" OR "bivariate regression" OR "statistical regression" OR "regression discontinuity*" OR "regression analysis" OR "statistical matching*" OR "propensity score matching" OR "covariate matching" OR "coarsened-exact matching" OR "propensity-weighted" OR inverse-probability-weighting OR "mixed method*" OR "cohort stud*" OR "cross sectional" OR "cross-sectional" OR "cohort analysis" OR "quantitative method*" OR "interrupted time series" OR ( before NEAR/5 after ) OR ( pre NEAR/5 post ) OR ( ( pretest OR "pre test" ) AND ( posttest OR "post test" ) ) OR ( "fixed effect*" NEAR/3 ( model OR estimation ) ) OR ( "random effect*" NEAR/3 ( model OR estimation ) ) OR ( ( quantitative OR "comparison group*" OR counterfactual OR "counter factual" OR counter-factual OR experiment* OR comparative ) NEAR/3 ( design OR study OR analysis)) OR (("semi-structured" OR semistructured OR prestructured OR pre-structured OR unstructured OR informal OR "in-depth" OR indepth OR "face-to-face" OR structured OR guide) NEAR/2 (interview* OR discussion* OR questionnaire*)) OR "focus group*" OR qualitative OR ethnograph* OR fieldwork OR "field work" OR "key informant" OR participatory OR "action research" OR "cooperative inquiry" OR "co-operative inquiry" OR case-stud* OR "case stud*" OR "community led" OR community-led OR barrier* OR facilitator* OR enabler* OR "panel data" OR (bivariate AND model) OR (multivariate AND model) OR (program* NEAR/3 (impact* OR assess*)) OR (gender* NEAR/3 (analysis OR approach*)) ) | 213,413 |
| (afghanistan OR albania OR algeria OR "american samoa" OR angola OR "antigua and barbuda" OR antigua OR barbuda OR argentina OR armenia OR armenian OR aruba OR azerbaijan OR bahrain OR bangladesh OR barbados OR belarus OR byelarus OR belorussia OR byelorussian OR belize OR "british honduras" OR benin OR dahomey OR bhutan OR bolivia OR "bosnia and herzegovina" OR bosnia OR herzegovina OR botswana OR bechuanaland OR brazil OR brasil OR bulgaria OR "burkina faso" OR "burkina fasso" OR "upper volta" OR burundi OR urundi OR "cabo verde" OR "cape verde" OR cambodia OR kampuchea OR "khmer republic" OR cameroon OR cameron OR cameroun OR "central african republic" OR "ubangi shari" OR chad OR chile OR china OR colombia OR comoros OR "comoro islands" OR "iles comores" OR mayotte OR "democratic republic of the congo" OR "democratic republic congo" OR congo OR zaire OR "costa rica" OR "cote d'ivoire" OR "cote d' ivoire" OR "cote divoire" OR "cote d ivoire" OR "ivory coast" OR croatia OR cuba OR cyprus OR "czech republic" OR czechoslovakia OR djibouti OR "french somaliland" OR dominica OR "dominican republic" OR ecuador OR egypt OR "united arab republic" OR "el salvador" OR "equatorial guinea" OR "spanish guinea" OR eritrea OR estonia OR eswatini OR swaziland OR ethiopia OR fiji OR gabon OR "gabonese republic" OR gambia OR "georgia (republic)" OR georgian OR ghana OR "gold coast" OR gibraltar OR greece OR grenada OR guam OR guatemala OR guinea OR "guinea bissau" OR guyana OR "british guiana" OR haiti OR hispaniola OR honduras OR hungary OR india OR indonesia OR timor OR iran OR iraq OR "isle of man" OR jamaica OR jordan OR kazakhstan OR kazakh OR kenya OR "democratic people's republic of korea" OR "republic of korea" OR "north korea" OR "south korea" OR korea OR kosovo OR kyrgyzstan OR kirghizia OR kirgizstan OR "kyrgyz republic" OR kirghiz OR laos OR "lao pdr" OR "lao people's democratic republic" OR latvia OR lebanon OR "lebanese republic" OR lesotho OR basutoland OR liberia OR libya OR "libyan arab jamahiriya" OR lithuania OR macau OR macao OR "macedonia (republic)" OR macedonia OR madagascar OR "malagasy republic" OR malawi OR nyasaland OR malaysia OR "malay federation" OR "malaya federation" OR maldives OR "indian ocean islands" OR "indian ocean" OR mali OR malta OR micronesia OR "federated states of micronesia" OR kiribati OR "marshall islands" OR nauru OR "northern mariana islands" OR palau OR tuvalu OR mauritania OR mauritius OR mexico OR moldova OR moldovian OR mongolia OR montenegro OR morocco OR ifni OR mozambique OR "portuguese east africa" OR myanmar OR burma OR namibia OR nepal OR "netherlands antilles" OR nicaragua OR niger OR nigeria OR oman OR muscat OR pakistan OR panama OR "papua new guinea" OR "new guinea" OR paraguay OR peru OR philippines OR philipines OR phillipines OR phillippines OR poland OR "polish people's republic" OR portugal OR "portuguese republic" OR "puerto rico" OR romania OR russia OR "russian federation" OR ussr OR "soviet union" OR "union of soviet socialist republics" OR rwanda OR ruanda OR samoa OR "pacific islands" OR polynesia OR "samoan islands" OR "navigator island" OR "navigator islands" OR "sao tome and principe" OR "saudi arabia" OR senegal OR serbia OR seychelles OR "sierra leone" OR slovakia OR "slovak republic" OR slovenia OR melanesia OR "solomon island" OR "solomon islands" OR "norfolk island" OR "norfolk islands" OR somalia OR "south africa" OR "south sudan" OR "sri lanka" OR ceylon OR "saint kitts and nevis" OR "st. kitts and nevis" OR "saint lucia" OR "st. lucia" OR "saint vincent and the grenadines" OR "saint vincent" OR "st. vincent" OR grenadines OR sudan OR suriname OR surinam OR "dutch guiana" OR "netherlands guiana" OR syria OR "syrian arab republic" OR tajikistan OR tadjikistan OR tadzhikistan OR tadzhik OR tanzania OR tanganyika OR thailand OR siam OR "timor leste" OR "east timor" OR togo OR "togolese republic" OR tonga OR "trinidad and tobago" OR trinidad OR tobago OR tunisia OR turkey OR "turkey (republic)" OR turkmenistan OR turkmen OR uganda OR ukraine OR uruguay OR uzbekistan OR uzbek OR vanuatu OR "new hebrides" OR venezuela OR vietnam OR "viet nam" OR "middle east" OR "west bank" OR gaza OR palestine OR yemen OR yugoslavia OR zambia OR zimbabwe OR "northern rhodesia" OR "global south" OR "africa south of the sahara" OR "sub-saharan africa" OR "subsaharan africa" OR "africa, central" OR "central africa" OR "africa, northern" OR "north africa" OR "northern africa" OR magreb OR maghrib OR sahara OR "africa, southern" OR "southern africa" OR "africa, eastern" OR "east africa" OR "eastern africa" OR "africa, western" OR "west africa" OR "western africa" OR "west indies" OR "indian ocean islands" OR caribbean OR "central america" OR "latin america" OR "south and central america" OR "south america" OR "asia, central" OR "central asia" OR "asia, northern" OR "north asia" OR "northern asia" OR "asia, southeastern" OR "southeastern asia" OR "south eastern asia" OR "southeast asia" OR "south east asia" OR "asia, western" OR "western asia" OR "europe, eastern" OR "east europe" OR "eastern europe" OR "developing country" OR "developing countries" OR "developing nation*" OR "developing population*" OR "developing world" OR "less developed countr*" OR "less developed nation*" OR "less developed population*" OR "less developed world" OR "lesser developed countr*" OR "lesser developed nation*" OR "lesser developed population*" OR "lesser developed world" OR "under developed countr*" OR "under developed nation*" OR "under developed population*" OR "under developed world" OR "underdeveloped countr*" OR "underdeveloped nation*" OR "underdeveloped population*" OR "underdeveloped world" OR "middle income countr*" OR "middle income nation*" OR "middle income population*" OR "low income countr*" OR "low income nation*" OR "low income population*" OR "lower income countr*" OR "lower income nation*" OR "lower income population*" OR "underserved countr*" OR "underserved nation*" OR "underserved population*" OR "underserved world" OR "under served countr*" OR "under served nation*" OR "under served population*" OR "under served world" OR "deprived countr*" OR "deprived nation*" OR "deprived population*" OR "deprived world" OR "poor countr*" OR "poor nation*" OR "poor population*" OR "poor world" OR "poorer countr*" OR "poorer nation*" OR "poorer population*" OR "poorer world" OR "developing econom*" OR "less developed econom*" OR "lesser developed econom*" OR "under developed econom*" OR "underdeveloped econom*" OR "middle income econom*" OR "low income econom*" OR "lower income econom*" OR "low gdp" OR "low gnp" OR "low gross domestic" OR "low gross national" OR "lower gdp" OR "lower gnp" OR "lower gross domestic" OR "lower gross national" OR lmic OR lmics OR "third world" OR "lami countr*" OR "transitional countr*" OR "emerging econom*" OR "emerging nation*") | 954,945 |

# Appendix B: Screening tool

**Screening tool for Systematic Review 4 (mechanisation and empowerment)**

1. Is the study conducted in low- and middle-income countries, as per the latest World

Bank Classification? If a multi-country study: are some of the countries considered low- and middle-income countries?

- Yes, include and see Question 2
- No, exclude on **country**

1. Does the study analyse impacts on rural populations?

- Yes, include and see Question 3
- No, exclude on **population**

1. Does the study evaluate a mechanisation intervention or analyse the impact of

mechanisation naturally occurring as the result of market forces? (see below for a

definition of ‘mechanisation’)

- Yes, include and see Question 4
- No, exclude on **intervention**

1. Does the study analyse population-level impacts of mechanisation such as changes in

cultivated areas, yields, labour, incomes, nutrition, access to health etc.?

- Yes, include and see Question 5
- No, exclude on **outcome**

1. Does the study have an experimental or non-experimental design with a valid

comparison group (including: regression discontinuity; interrupted time series;

difference-in-differences; propensity score matching and other matching methods;

endogenous switching regression; control function approach; instrumental variables;

Roy’s model; Heckman’s selection correction; panel fixed effects models)?

- Yes, include for effectiveness and Stop!
- No, see Question 6

1. Does the study have a simulation design (such as microsimulation; computable general

equilibrium model; structural modelling; input-output model)?

- Yes, include among ‘simulation studies’ and Stop!
- No, see Question 7

1. Does the study have cross-country, cross-state, or cross-region design (such as a

panel fixed-effects analysis of several countries, or of states and regions within a

country; and synthetic control analysis)?

- Yes, include among ‘comparative analysis studies’ and Stop!
- No, see Question 8

1. Does the study identify the implementation issues of mechanisation or mechanisation

interventions **on women** using qualitative methods?

- Yes, include among ‘process evaluation and qualitative studies’
- No, exclude on **design**

**Definition of mechanisation and mechanisation interventions**

We define mechanisation as the adoption of tools and machinery in agricultural production (e.g., ploughs, tractors, carts, threshers, harvesters, planters, winnowers, grinders). Mechanisation also includes the transport of agricultural inputs and outputs during the production process (such as transportation of fertiliser to the farm or produce to a storage facility), and irrigation operated through mechanical and powered pumps.

Mechanisation is a market outcome. Some farmers purchase or rent and use tools and machinery in agricultural production. However, government agencies and NGO can also promote mechanisation in various ways through mechanisation interventions, which may consist of direct provision of tools and machines; incentives to acquiring machines (e.g., credit, subsidised imports); information on the positive effects of mechanisation; and training extension on the use of agricultural machinery. Governments can also promote mechanisation by changing the environment through investments in mechanisation research and design, legal frameworks on patents and rental contracts, education of engineers and specialised schools, and information campaigns.

# Appendix C: Data extraction tool

| **CODING TOOLS** | |
| --- | --- |
| Category | Subcategory |
| **Publication status** | - Ongoing - Completed |
| **Region** | - East Asia & Pacific - Europe & Central Asia - Latin America & Caribbean - Middle East & North Africa - South Asia - Sub-Saharan Africa |
| **Country name** |  |
| **Settings** | - Rural - Urban - Rural and urban (both) - Not clear |
| **Project/intervention name** |  |
| **Year** |  |
| **Funding agency** |  |
| **Gender** | - Male - Female - Non-binary - All sexes - Not reported |
| **BAME** | - Mainly/exclusively (80%+) - Partly - None - Not clear |
| **Study design** | - Experimental design - Non-experimental design - Process evaluation |
| **Study method** | - Randomised controlled trial - Difference in difference - Instrumental variable estimation - Regression discontinuity design - Statistical matching - Interrupted time series - Fixed effects estimation - Other regression |
| **Mixed method** | - Yes - No |
| **Intervention category** | **Intervention subcategory** |
| No intervention/market forces ((replacement of human or animal work with mechanical tools)) | - No intervention/market forces |
| Intervention promotes the use of mechanised tools | - Subsidized provision of mechanical tools - Hiring services |
| Interventions focus on Promotion | - Promotion: training and extension - Promotion: fuel/energy subsidies - Promotion: loans - Promotion: behavioural change |
| Enabling environment | - Enabling environment |
|  |  |
| **Outcome domain** | **Outcome sub-domain** |
| Empowerment | - Indices of economic empowerment - Agency indicators of decision-making on use of resources - Psychological tests of autonomy and agency - Achievement indicators such as leadership positions in groups, ability to make economic decisions, access to markets, bargaining power, and control over resources |
| Health effects | - Disability - Healthcare-seeking behaviour - Health outcomes - Child nutrition |
| Economic effects | - Labour productivity - Use of own and hired labour - Cultivated area and yields - Time use and drudgery - Income and expenditure |
| Participation | - Participation in interventions - Group membership - Technology adoption |
| Attrition |  |
| Differential attrition |  |
| Barriers and facilitators to participation |  |
| Barriers and facilitators to outcome |  |
| Design issues |  |
| Implementation issues |  |
| What target populations say |  |
| Moderators and confounders |  |

## Appendix D: Definition of the intervention

|  | Intervention category | Intervention subcategory | Definition |
| --- | --- | --- | --- |
|  | No intervention/market forces | No intervention/market forces | When there is no public intervention, the paper is just looking at impact of mechanisation has it happens |
|  | Intervention promotes the use of mechanised tools | Subsidized provision of mechanical tools | When the gov or project provides subsidies to purchase machines, note this includes machines donations (100% subsidy) |
|  |  | Hiring services | When private firms, informal networks, cooperatives, projects etc. establish mechanisms to allow farmers to hire machines from centres |
|  | Interventions focus on Promotion | Promotion: training and extension | When project promotes mechanisation by training and extension |
|  |  | Promotion: fuel/energy subsidies: | When project promotes mechanisation through subsidised energy |
|  |  | Promotion loans | When project promotes mechanisation through credit access |
|  |  | Promotion behavioural change | When project promotes mechanisation by changing attitudes, promoting their effectiveness etc |
|  | Enabling environment |  | When the government promotes research and development, changes laws, trade policies etc. |
